# WHIRL Study: Workplace Health Interprofessional Learning in the Construction Industry

**DOI:** 10.3390/ijerph17186815

**Published:** 2020-09-18

**Authors:** Holly Blake, Sarah Somerset, Katharine Whittingham, Matthew Middleton, Mehmet Yildirim, Catrin Evans

**Affiliations:** 1 School of Health Sciences, University of Nottingham, Nottingham NG7 2HA, UK; sarah.somerset@nottingham.ac.uk (S.S.); katharine.whittingham@nottingham.ac.uk (K.W.); ntxmy4@exmail.nottingham.ac.uk (M.Y.); catrin.evans@nottingham.ac.uk (C.E.); 2 National Institute for Health Research (NIHR) Nottingham Biomedical Research Centre, Nottingham NG7 2UH, UK; 3 School of Medicine, University of Nottingham, Nottingham NG7 2UH, UK; mzymm16@exmail.nottingham.ac.uk

**Keywords:** workplace, health promotion, interprofessional learning, interprofessional education, construction, public health, health checks

## Abstract

Interprofessional learning (IPL) is essential to prepare healthcare trainees as the future public health workforce. WHIRL (Workplace Health InteRprofessional Learning) was an innovative IPL intervention that engaged volunteer healthcare trainees (*n* = 20) in multi-professional teams to deliver health checks (*n* = 464), including tailored advice and signposting, to employees in the UK construction industry (across 21 events, 16 sites, 10 organisations) as part of an ongoing research programme called Test@Work. Volunteers undertook a four-part training and support package of trainer-led education, observations of practice, self-directed learning and clinical supervision, together with peer mentoring. In a one-group post-test only design, IPL outcomes were measured using the Inventory of Reflective Vignette-Interprofessional Learning (IRV-IPL), and the psychometric properties of the IRV-IPL tool were tested. WHIRL demonstrably improved healthcare trainees’ interprofessional skills in all five areas of collaboration, coordination, cooperation, communication, and commendation. The IRV-IPL tool was found to be a valid and reliable measure of interprofessional competencies across three scenarios; before and after health promotion activities, and as a predictor of future health promotion competence. This industry-based workplace IPL programme resulted in the attainment of health check competencies and bridged the gap between research, education and clinical practice.

## 1. Introduction

Interprofessional collaboration in education and practice is advocated as an innovative strategy that will play an important role in mitigating the global health workforce crisis [1]. This paper presents a public health interprofessional learning (IPL) project embedded within the context of a workplace health promotion research study, in which healthcare students and staff collaborated in the delivery of health checks in a “real-world” industry workplace setting.

IPL takes place when individuals from different professional backgrounds actively engage together to develop the skills necessary to collaborate successfully [1]. IPL is commonly advocated in the context of health professions education, on the understanding that health professional students can most effectively acquire the knowledge, skills and attitudes required to promote health as part of an effective team when they learn and practice together with individuals from different professions or specialities [2,3,4]. IPL has been defined by the United Kingdom (UK) Centre for the Advancement of Interprofessional Education as a situation where “two or more professions learn with, from, and about each other, to improve collaboration and the quality of care”. This process is essential to prepare healthcare graduates with the knowledge, skills, and attitudes to engage in effective interprofessional collaboration in practice. Likewise, healthcare education programmes must meet the professional requirements set out by regulatory bodies to prepare healthcare students to achieve interprofessional collaboration competencies. Intentionally integrating learning opportunities into health professions education is therefore important to adequately prepare a “collaborative-ready” healthcare workforce [5].

Interprofessional education provides opportunities for health professional learners to engage in IPL. Examples include engagement in shared educational curricula, modules or short courses, e.g., [6,7,8], or shared conference events, e.g., [9]. Alternatively, it may involve face-to-face interactive opportunities; these are most often focused within clinical environments and may entail collaborative working during student placements in clinical settings [10,11], assessment of clinical skills in a team situation [12]: Team Objective Structured Clinical Examination; OSCE), engagement in clinical simulated experiences [13,14,15], or consideration of clinically-focused case studies or scenarios [16,17,18]. 

IPL activities usually take place in clinical settings, such as hospital wards [10,11,19] or community healthcare settings (rehabilitation: [20], primary care: [21]. There are far fewer published reports of interprofessional learning activities taking place outside of clinical settings. However, interprofessional health promotion activities have been undertaken with some success in schools [22]: cookery project), higher education institutions [9]: health conference, [23]: rural university sports, senior wellness fairs [24]: healthy aging and social care settings [6]: cardiovascular disease prevention for older adults. To our knowledge, there are no existing interprofessional learning projects for healthcare students in the non-clinical workplace setting, despite workplaces continuing to be a key focus for the promotion of population health [25]. The workplace setting is the focus of this paper.

The workplace is becoming increasingly popular as a setting for promoting public health through health promotion initiatives. Such initiatives might include the promotion of health behaviours (e.g., diet, physical activity, smoking cessation) or the provision of general health checks for employees. The exponential rise in the prevalence of chronic conditions associated with unhealthy lifestyles means that the acquisition of skills in health promotion practice is becoming essential for all healthcare practitioners. In the UK, the national initiative “Making Every Contact Count” (MECC: https://stpsupport.nice.org.uk/mecc/index.html) advocates that health and care workers should endeavour to increase the support available to help people to manage and improve their own health and wellbeing, through brief interventions. One way to achieve this is to engage people in “*conversations about improving their health by addressing risk factors such as alcohol, diet, physical activity, smoking and mental wellbeing*” (MECC). In the workplace setting, this national public health initiative can be supported through the provision of general health checks to employees, which provides an opportunity to engage large numbers of working-age adults in conversations about their health and lifestyle behaviours. 

Since engagement in health promotion is a key expectation of all healthcare professionals, promoting health through the workplace setting provides vital opportunities for health promotion practice. For trainee healthcare professionals, engagement in workplace health promotion provides not only IPL opportunities, but will also assist in building a public health workforce for the future who have “real-world” experience of “making every contact count” in promoting public health. Research has demonstrated the benefits of promoting health through the workplace for employees and employers [26,27,28,29,30,31,32,33]. Although large organisations are more likely to participate in workplace health promotion initiatives due to scale, space and resources, there is an ongoing need to encourage the engagement of small businesses in workforce health initiatives more broadly [34]. Specifically, general health checks in the workplace have shown to be well-received when delivered [35,36], although they are rarely included in corporate health and wellness programmes in UK organisations of any size [37].

IPL is strongly advocated in healthcare education, and healthcare practice. In addition, interprofessional collaboration is standard practice within the vast majority of research [38]. Research teams are increasingly multi-professional and inter-disciplinary and aspiring researchers require confidence and competence to work in such diverse disciplinary environments. However, there appear to be missed opportunities (within both healthcare education and healthcare practice) to provide students with opportunities to access IPL in the context of research. Efforts to bridge the gap between research, education and clinical practice may be fruitful with regards to increasing IPL opportunities for the next generation of healthcare professionals. This paper reports an innovative project in which this gap was bridged through the development, delivery and evaluation of an IPL health promotion initiative, which is set within the context of a workplace research study in the industry.

### Project Description and Aims

The innovative IPL initiative reported in this paper is entitled “WHIRL” (Workplace Health InteRprofessional Learning). It is an IPL project embedded within a workplace health promotion research study called Test@Work. The Test@Work study is a project in which employees in the construction industry are offered HIV testing in the workplace as part of a multi-component health check. This research project, therefore, generates opportunities for health promotion practice in small, medium and large organisations (https://www.theconstructionindex.co.uk/news/view/breaking-the-hiv-taboo). WHIRL provides IPL opportunities for healthcare student volunteers to engage with healthcare professionals and other students from diverse disciplines through the team delivery of the Test@Work health checks in an industry workplace setting. In this way, WHIRL bridges the gap between research, education and practice by providing IPL opportunities within the context of multi-professional team health check delivery, delivered as health promotion activity as part of a research study in a “real-world” non-clinical setting. For the purpose of this study, multi-professional is defined as activities involving three or more professional groups.

The aim of the WHIRL IPL initiative was to:

- provide a research-based IPL opportunity for healthcare students to engage in “real-world” health promotion within an inter-professional team in a workplace setting.
- the learning objective was to provide knowledge, skills, and values to be taken forwards into future healthcare practice.

The aim of this research was to:

- describe the WHIRL initiative with regards to innovative alignment to research and industry, volunteer recruitment, training and supervision, workplace health promotion activity, attainment of skills and competencies.
- assess the interprofessional learning outcomes for WHIRL IPL volunteers using the Inventory of Reflective Vignette-Interprofessional Learning (IRV-IPL).
- test the reliability and validity of the IRV-IPL for use with healthcare students in “real-world” health promotion settings.

## 2. Methods 

### 2.1. Study Design

A one-group post-test only design was adopted which was appropriate to the contextual circumstances of the study. In this design, the effect of participation in the WHIRL initiative on a range of potential IPL outcomes was measured after a single large group of volunteer participants had engaged in a predetermined activity (in this case, team delivery of health checks). We adopted an approach used by Ong et al. [39], in which the “Inventory of Reflective Vignette” (IRV) was used as a framework, and the pre-test items were embedded into a post-test survey to allow for reflection on prior and current conditions, and minimise possible bias for health check delivery performance [40]. This strategy has been used previously [40,41]. 

### 2.2. Participants

Participants in the WHIRL IPL initiative were referred to as IPL volunteers. This is because the project team referred to the construction site employees receiving the Test@Work health checks as “participants” (in the research programme), and the WHIRL participants individually as “IPL volunteers”, and collectively as “the health promotion team” or the “IPL team”. The IPL volunteers included healthcare students from a single higher education institution in the UK. The only pre-requisite was that volunteers expressed an interest in engaging with health promotion activities in a “real-world”, non-clinical setting. There were no other specific inclusion or exclusion criteria. 

### 2.3. Setting and Target Audience

The health check recipients were employees from the construction industry, of any occupation, job contract or level. Ten participating organisations (“employers”) were from the private sector with sites in the UK employing construction workers and hosting a total of 4649 staff (9 large, 1 small-to-medium enterprise: SME). These organisations were recruited as part of the Test@Work study. Further details about the host organisations and their employees, data on employee engagement, and perceptions of the Test@Work health checks are reported elsewhere. 

### 2.4. Volunteer Training and Supervision

Volunteers were equipped with the skills to deliver workplace health checks and provide tailored advice to clients with appropriate signposting through a combination of three strategies: (a) trainer-led sessions (face-to-face) and observation of practice; (b) self-directed training; and (c) clinical supervision. Skills for undertaking diagnostic tests following onward referral of at-risk people (e.g., clinical follow-up) were not required as these tests and follow-on care were beyond the scope of the health check. 

#### 2.4.1. Trainer-Led Sessions and Observation of Practice

Prior to health check delivery, all volunteers received general information about the Test@Work health checks from the study team and attended a face-to-face training session (duration: 60–120 min); session length was dependent on participant’s prior training, level of knowledge and experience. The training session comprised of education about health promotion and behaviour change, discussion and interactive practical demonstrations including a minimum of one observation of practice, and one observed attempt. Trainer led sessions and demonstrations were delivered by a cardiac nurse with a special interest in health promotion, a paramedic and a health promotion coordinator. The health checks included confidential measures of weight, height, and calculation of body mass index (BMI), waist and waist-to-hip ratio measurements, blood pressure and a screening test for mental wellbeing. For each assessment, IPL volunteers were shown: (i) how to use associated equipment and measures, (ii) how to interpret health results, (iii) how to give brief tailored health advice to health check recipients, including signposting participants to relevant services if there were any concerns following the health check (e.g., general practitioner).

#### 2.4.2. Self-Directed Training

In advance of the health check, all IPL volunteers were required to engage in additional self-directed training which included a training slide set, and an information pack based on Making Every Contact Count (MECC) resources (https://www.gov.uk/government/publications/making-every-contact-count-mecc-practical-resources) with health leaflets and guidance around diabetes, heart health, physical activity and diet, musculoskeletal health and mental wellbeing.

#### 2.4.3. Clinical Supervision

Clinical supervision was delivered by a cardiac nurse “trainer” with significant expertise in health promotion practice, and public health education, and a health promotion co-ordinator with a specialist interest in workplace health. The trainer was competent in all areas of the core, clinical skills and the NHS Health Check programme competencies. During the event, the IPL volunteers had access to an experienced clinical member of the project team who coordinated and oversaw activities and acted as the main point of contact as a clinical supervisor. This ensured quality in the delivery of health checks since IPL volunteers had ongoing supervision, training opportunities and support. The more experienced volunteers became peer mentors, supporting those who were new to health promotion practice. The peer mentors (*n* = 3) included two healthcare students who were qualified nurses and one who was a paramedic.

#### 2.4.4. Volunteer Competencies

The training was informed by the NHS Health Check Competency Framework [42]: https://www.healthcheck.nhs.uk/commissioners-and-providers/national-guidance/. These competencies are based on National Occupational Standards which describe the skills, knowledge and understanding needed to undertake a particular task or job to a nationally recognised level of competence. The Test@Work health checks incorporated some elements of the NHS Health Checks (specifically cardiovascular and diabetes risk and associated lifestyle advice) although the Test@Work checks also included a mental wellbeing screening assessment and advice around musculoskeletal health. The training covered core competencies required for delivery of NHS Health Checks (e.g., effective communication; equality, diversity and inclusion; duty of care; safeguarding; person-centred care; keeping records and handling information, infection prevention; privacy and dignity). As registered students, all volunteers were provided with health and safety training by their institution. Involvement as IPL volunteers and completing data collection tools for the WHIRL study included a reflection on their own role, and their own personal development. The practical application of the Test@Work health checks is mapped to competencies and draws on elements of the Skills for Health competency tools provided by Public Health England for use in the NHS Health Checks (Table 1). 

Registered healthcare professionals had their own codes of practice that they adhered to, although all IPL volunteers carrying out the health checks were encouraged to work in line with the Code of Conduct for Healthcare Support Workers and Adult Social Care Workers (https://www.skillsforhealth.org.uk/standards/item/217-code-of-conduct). The code clearly describes the standards of conduct, behaviour and attitudes that are expected of workers providing care and support.

### 2.5. Assessment of Inter-Professional Learning (IPL)

The Kirkpatrick Four-Level Evaluation Model [43] is widely accepted to be one of the most commonly used industry-standard methods to evaluate the effectiveness of learning solutions. This model was used to evaluate the WHIRL IPL project. This included evaluations at four levels including: Level 1: ‘Reaction to the WHIRL IPL activity’–this is the degree to which volunteers were attracted to the IPL opportunity and found it relevant to their healthcare training (e.g., evidenced by expressions of interest in volunteering: the number of students engaged, disciplines involved, diversity in volunteer pool); Level 2: ‘Learning against agreed learning outcomes’–this is the degree to which volunteers acquired the intended knowledge, skills, attitude, confidence and commitment based on their participation in WHIRL IPL (e.g., evidenced by IPL competencies before and after engagement, and in a hypothetical future scenario-as assessed by the Inventory of Reflective Vignette–Interprofessional Learning); Level 3: ‘How behaviour has changed as a result of the learning’–this is the degree to which the students engaged in the health promotion (e.g., evidenced by observation (as either observer and/or practitioner) and application of this learning in health check delivery); Level 4: ‘How the learning has impacted on business or environmental outcomes’–this is the extent to which the IPL activities reached the target industry, and how employees engaged with the provision (e.g., evidenced by the number of organisations and host sites involved and the number of health checks successfully delivered to employees in the construction industry). 

For Level 2, the Inventory of Reflective Vignette–Interprofessional Learning (IRV-IPL) was used to assess IPL competency outcomes (Supplementary Materials Table S2). The IRV-IPL is a valid and reliable reflective tool to assess IPL as a Continuing Professional Development (CPD) outcome [39]. The IRV-IPL has demonstrated beneficial measures for *postdictive* validity in recalling prior interprofessional competencies, and *predictive* validity in estimating IPL as an outcome of CPD and alternative interventions [39]. 

The IRV-IPL assesses five items in each of the following areas: collaboration, coordination, cooperation, communication, and commendation. The measure has three segments to assess interprofessional competencies “before”, “after”, and “what if” scenarios using vignettes. This tool was selected since it exhibits good psychometric properties with high internal consistency, and evidenced suitability of length, validity of content, practicality of administration, inclusivity of user, usability of tool, and clarity of structure [39]. The original authors found the IRV-IPL generated excellent internal consistency (α = 0.98), and across all segments of collaboration (α = 0.96), coordination (α = 0.96), cooperation (α = 0.96), communication (α = 0.97), and commendation (α = 0.98). The instrument is divided into two columns, one for the assessment items and another for the rating responses. Responses are given on a 6-point Likert-type scale (i.e., 1 = Emerging; 2 = Developing; 3 = Minimal; 4 = Proficient; 5 = Advanced; 6 = Excellent) which is intended to allow deeper reflection yet eliminate a neutral value for clearer measurement. Tool constructs and how they were applied within the WHIRL initiative are provided in Table 2. 

The vignettes in the IRV-IPL were developed by Ong et al. [39], guided by a five-step tool development procedure outlined by Ruzafa-Martinez and colleagues [44], comprised of (1) literature review and existing tool synthesis, (2) expert content validation, (3) pilot testing, (4) reliability and validity testing and, (5) implementation with validation and analysis. Here, the original vignettes were used although the term “participants” was replaced by “team members” to enhance clarity. 

### 2.6. Study Procedure

WHIRL IPL data were collected in February–March 2020, data collection was undertaken by two members of the project team (SS, HB). All IPL volunteers were required to complete the IRV-IPL. This was completed at the end of their involvement with the WHIRL study.

#### 2.6.1. Ethical Approval

The local institutional ethics review committee approved the study (Ref: LT12042016). Site-level consent was obtained from organisational representatives by the Test@Work team. At an individual level, all health check recipients signed consent forms, and all IPL volunteers signed a procedural and data protection agreement form.

#### 2.6.2. IPL Volunteer Recruitment

The project team circulated information about the IPL opportunity via institutional bulletins and magazines, educational course leaders, and direct emails. Interested students or staff then contacted the project lead or project researcher to join the WHIRL team. The project involved a rolling recruitment and training process from August 2019 to February 2020. The sample characteristics are reported in Section 3.1.

#### 2.6.3. Health Check Procedure

Interpersonal contact is deemed to be an effective way to increase inter-group understanding, promote positive intergroup attitudes and reduce prejudice between individuals from different groups [45,46]. Therefore, Adult Learning Theory and Intergroup Contact Hypothesis [47] was used as a guiding framework to inform the alignment of the WHIRL IPL initiative with the Test@Work health check delivery plan as described below. Specifically, to determine the contributions, roles and expectations of each individual within the inter-professional volunteer group. 

Health checks were delivered by a small inter-professional team comprised of volunteers and staff, determined by their availability. Each event was therefore delivered by a different team. Individual IPL volunteers within the team were required to deliver specific elements only, but to work in collaboration with the other IPL volunteers with regards timings, rotation of activities where required and coordination of activities. First-line calibration on clinical equipment was undertaken in the first instance by the attending clinical staff member or the health promotion co-ordinator, and then by supervised IPL volunteers once they were proficient at health check set-up. Each volunteer was responsible for one or two elements of the health check, as agreed with their event team at the start of each event day. Individuals kept their own records, which were transferred to the project coordinator at the event end. The IPL volunteers were required to undertake their allocated checks and then provide brief tailored health advice and signposting relating to cardiac and diabetes risk, weight management, lifestyle behaviour (diet and physical activity), musculoskeletal health, stress and mental health awareness. Tailored advice was guided by the Making Every Contact Count (MECC) Implementation Guide and Toolkit which is freely available online at: (https://learning.wm.hee.nhs.uk/sites/default/files/behaviour_change_care_pathway_and_competence_mapping_0.pdf). This guide was followed with the exception of the suggestion to provide further follow-up due to the one-off nature of the checks in this study. The NHS Health Checks StARS framework was adapted for this context (Supplementary Materials Table S1), which ensured that a systems approach was adopted with the involvement of key internal and external partners at the heart of the process. Content and delivery were therefore based on advice and standards from existing national guidance. This framework brings together criteria into ten themes from leadership and planning to commissioning and the delivery of risk assessment and management (https://www.healthcheck.nhs.uk/commissioners-and-providers/delivery/nhs-health-check-stars-framework/). 

Any individual working at the host site on the day of the health check event was eligible to take part, including employees, self-employed and agency workers (herein referred to as “employees” in the context of the host site, or “clients” in the context of participation in the health check). There were no exclusions set by the project team. Clients booked an appointment time in advance of the event, via the coordinating staff member from the host organisation, who provided the Test@Work team with an event booking list. Employees at the participating organisation were also able to attend without booking as drop-ins were available during the day. Clients that booked but missed their appointments were able to attend at an alternative time. In instances where there was appointment availability on the day, members of the Test@Work project team actively promoted the opportunity in communal areas. 

Clients were initially greeted by a member of the Test@Work team who provided them with information about the health check, took informed consent and gave them a personal health record sheet and a take-away resource pack of health information. Clients were then signposted to the WHIRL IPL volunteers who conducted the check and recorded their results on the personal record sheet held by the client. Volunteers then provided tailored health advice using the client’s results in conjunction with the resource pack. Once the general health check was completed, clients that had opted for the HIV test were signposted by WHIRL volunteers to the sexual health service partners in another private room. The entire health check service was provided to clients free of charge, and individuals could choose from one or all of the available health checks and tests and/or engage in discussion related to the health information provided in their take-away resource packs. No health data were stored by the project team or provided to the host organisations, clients held their own data record and took it away with them.

A pragmatic approach was adopted to ensure that the IPL opportunity reflected the way in which workplace health provision would likely operate in standard practice. Therefore, the time individual IPL volunteers spent with a client for their brief intervention varied from 5–15 min depending on the checks selected by the recipient, and the nature of the discussion. The size of the team and the total number of team members present at each event varied depending on the number of employees onsite at the participating organisation (min = 3, max = 10, average = 7). Between two and five IPL volunteers delivered the health checks at each event. Event volunteers were recruited from the wider WHIRL IPL team (according to their availability for the arranged date and time for the event). The IPL volunteers were therefore working alongside other IPL volunteers from a range of professions, and the combination of professions differed between events. The health check events took place in dedicated private spaces within office complexes (*n* = 7) and building sites (*n* = 9).

Opt-in HIV testing was available to clients at the same health check events; this element was delivered in a separate private area by experienced sexual health advisors from a third-party organisation, reporting to and overseen by a UK local clinical commissioning group. Key contacts from the participating organisations were sent a digital toolkit see [48] prior to the event with information and guidance around workplace health promotion, health checks and HIV testing. This employer toolkit contained information about employee and employer responsibilities should an employee choose to disclose a positive test result to their employer.

The IPL volunteers were provided with information and guidance on workplace HIV testing to ensure they understood the overall context of the event and had access to the employer toolkit, but they were not involved in delivery of the sexual health consultations, HIV testing, discussions with employees about their test results or sign-posting to HIV-specific follow-up. The inclusion of HIV testing within the health checks required that IPL volunteers were required to coordinate and liaise with the project team and a clinical supervisor, the other IPL volunteers, and third-party delivery partners to ensure smooth delivery of the health checks for clients at the participating organisations.

### 2.7. Data Analysis

Data were analysed using IBM SPSS Statistics version 24 IBM SPSS Statistics for Windows, Version 24.0. (IBM Corp, Armonk, NY, USA). The internal reliability of the IRV-IPL was determined using Cronbach’s alpha and validity coefficient using the item-total correlation. We applied the Pearson Correlation method for the correlation matrix. For this, we used total scores of each construct and total scores of Before, After and What if. Mean and standard deviation (s.d.) are presented for IRV-IPL test responses.

## 3. Results

The findings are mapped to the Kirkpatrick Four-Level Evaluation Model [43], which is one of the most commonly used models for evaluating training and educational programmes.

### 3.1. Reaction to the WHIRL IPL Activity (Level 1)

“Reaction” in this context refers to both the uptake (and group diversity) as well as the experiences of IPL volunteers. This IPL opportunity engaged a diverse group of 20 healthcare students working with a further six staff IPL event contributors at 21 events, with a different team (staff-student combination) at each event. Staff contributors included three registered nurses, one health psychologist and one physiotherapist. IPL volunteers were registered students across five healthcare disciplines from a single institution in the UK (medicine, *n* = 1, nursing, *n* = 15, physiotherapy, *n* = 2, and health psychology, *n* = 2). There were seven pre-registered healthcare students (35%: two undergraduate bachelors, five graduate-entry Bachelor’s courses), and 13 postgraduates (65%: seven Master’s level, and six doctoral level courses). In addition to diversity in discipline, they had varying levels of clinical experience providing a unique opportunity for team learning; some had only observed clinical skills, some had experience of clinical practice but not specifically of delivering health checks, and some were registered healthcare professionals who had prior experience of health promotion practice and were undertaking further study (e.g., care assistant, registered nurse, paramedic). IPL volunteers were 11 females (F, 55%) and 9 males (M, 45%) (13/65% from black and minority ethnic groups (BAME), of which 6F/46%, 7M/54%). Due to variations in the size of sites and the number of employees at each site, the size of the IPL volunteer team varied at each event. However, the team always included at least two different professions, and at least one healthcare professional experienced in health promotion practice. Each IPL volunteer delivered health checks at between one and six site events (as part of a different team on each occasion), although the majority delivered at two to three events. The actual number of health checks delivered by any individual IPL volunteer, therefore, ranged from 12 to 98, although the average was 32.

A detailed evaluation of the health check events is reported elsewhere. Post-event feedback indicated that all 20 volunteers enjoyed their experience and found it useful for their learning and practice (100%). They found the training helpful (particularly those with less prior experience) and reported that the resources provided helped with interpreting health check results and supported individualised health conversations. They were overwhelmingly positive towards the opportunity for interactions with students and staff from other disciplines, across multiple year groups and healthcare courses. Those who were already registered healthcare professionals enjoyed being able to demonstrate their skills in a new setting and support less experienced peers. Likewise, those newer to health promotion practice were positive towards the team support from peers as well as the wider project team and clinical supervisor(s). 

### 3.2. Learning against Agreed Learning Outcomes (Level 2)

One hundred percent of volunteers completed the IRV-IPL self-assessment. As shown in Table 3, the mean responses of participants on the IPL items before the health checks ranged from two (Developing) to five (Advanced) depending on the prior expertise of the volunteer. Students who were already registered healthcare professionals (e.g., nurse or paramedic) reported Proficient or Advanced level skills at the outset, whereas those who were not registered healthcare professionals reported Emerging or Developing skills prior to the health checks. IPL scores after the health checks ranged from four (Proficient) to six (Excellent), again with registered healthcare professionals rating their skills as Advanced or Excellent, whereas those who were not registered healthcare professionals rated their skills as Proficient or Advanced. When asked to consider their perception of future delivery (“If…health promotion event”), ratings were mostly Proficient or Advanced for those who were not registered healthcare professionals, but Advanced or Excellent for those who were registered. There was an improvement in scores across scenarios for every single item on the IRV-IPL. Specifically, there was a significant linear increase in overall IRV-IPL scores (from before, to after) for 100% of the IPL volunteers, with these positive values either sustained or further increased when considering the future scenario. The vast majority of items in the “What If” scenario were rated as Advanced or Excellent. Moreover, there are statistically significant increases between Before, After, and What If constructs’ mean scores as shown in Table 4.

#### Test for Reliability and Validity

The IRV-IPL demonstrated excellent internal consistency (overall α = 0.97) across all segments, with overall reliability estimates remaining broadly stable as “excellent” across the three scenarios which suggests that the tool was able to measure the five constructs consistently (Table 5): before (α = 0.96), after (α = 0.97), and if (α = 0.96). Each constructs’ reliability was calculated based on their five items, and the overall scores were calculated depending on all relevant items. Each of the five identified constructs and overall scores showed high internal reliability: collaboration (α = 0.92), coordination (α = 0.87), cooperation (α = 0.94), communication (α = 0.92) and commendation (α = 0.96). The tool was therefore deemed to serve as a reliable measure of interprofessional learning in this workplace health promotion context.

Furthermore, Pearson correlation analysis with total scores of each construct versus total scores of before, after and what if is applied (Table 6). This indicates a strong significant positive relationship (r > 0.70, *p* < 0.05) across all IPL constructs when before and after were correlated, with the exception of communication (r < 0.70, *p* < 0.05). This may imply the distinctness of this skill in healthcare disciplines, since communication and reflection are distinct and core elements of healthcare educational programmes. Analysis of the degree of correlation between (a) before and what if, and (b) after and what if scores showed a significant positive limited relationship (r < 0.70, *p* < 0.05) in all IPL constructs.

Content and face validity of IRV-IPL was previously demonstrated by the developers [39], but was further confirmed in this study by expert review by university lecturers (*n* = 5) with an interest in IPL. Statistical validity was tested by correlating each item with the total score (i.e., the sum of all segments). The actual validity coefficients (Table 7) of the corresponding items for all constructs consistently exhibited significant positive correlations (r > 0.35, *p* < 0.05) in all segments, with the exception of one item: “Inform other participants for any changes”. For this item, almost no relationship was observed between the statement and “before”, “after”, and “what if” total scores. However, it should be noted that this item had to lowest score at the outset (perhaps indicating an expectation that changes would always be communicated by project leads), and the highest score after the intervention (perhaps indicating newfound confidence to lead on communicating to others) and therefore this item showed the greatest change (improvement) between before and after scores compared to any other item. The findings overall demonstrate the validity of the IRV-IPL as an assessment tool and show that the instrument is able to measure interprofessional learning across each segment. The IRV-IPL has demonstrated it is able to determine both postdictive validity (recall of prior interprofessional competency) and predictive validity (estimating IPL as an outcome of future interventions). The tool was therefore deemed to serve as a valid measure of interprofessional learning in a workplace health promotion context. 

Overall, the results suggest that IRV-IPL can provide reliable and valid reflective assessments of (a) baseline interprofessional competencies, (b) IPL as outcomes of an intervention, and (c) comparative IPL measure for alternative situations. 

### 3.3. How Behaviour Has Changed as a Result of the Learning (Level 3)

During the study, 100% of IPL volunteers completed observations and/or demonstrated practice to new volunteers as determined by prior experience, and all of them successfully delivered health checks. The “What If” predictive measure in the IRV-IPL is indicative of the perceived future application of learning for all the volunteers. According to the Kirkpatrick model, application of learning is best assessed 3–6 months after training, therefore evaluation comments made by students who were recruited into the WHIRL team in months 1–4 could include a reflection on how they had actually applied their learning to health promotion practice (more broadly) after a minimum of three months. A commonly reported learning point was a greater understanding of the health inequalities and the health needs of particular populations (e.g., this industry included construction workers, low waged manual workers, migrant workers, workers in the gig economy, and individuals who may engage in risky health behaviours). Areas of learning application for IPL volunteers included increased confidence in health promotion practice, as well as an increased desire to promote health in non-clinical settings and actively seeking out opportunities to engage in brief health promotion interventions (e.g., initiate more MECC discussions). 

### 3.4. How the Learning Has Impacted on Business or Environmental Outcomes (Level 4)

This project successfully engaged employers. Over 7 months, between 15 August 2019 and 11 March 2020, 10 organisations from the construction industry took part, including one SME (seldom reached by employee wellness programmes). The workplace health check events contributed to the employee health and wellbeing strategies at the participating organisations and for the SME it was their only employee health provision. The WHIRL IPL team successfully delivered 21 health check events for these 10 organisations, across 16 worksites, reaching a total of 464 employees. All the participating organisations engaged with the employer toolkit (100%) which provided training around workplace health promotion, health screening and HIV testing. Detailed event and employer toolkit evaluation, including the impacts of the health checks on (and views of) employees and employers, are reported elsewhere.

## 4. Discussion

To our knowledge, this is the first theoretically informed IPL initiative to be delivered in a “real-world” non-clinical workplace setting, bridging the gap between research, education and health promotion practice. The WHIRL IPL project successfully engaged 21 “teams” of healthcare trainees and healthcare professionals working with public sector and third sector partners, to deliver health check events within the UK construction industry, across 21 events, at 16 sites of 10 SMEs and large organisations. Our volunteer group was multi-professional, inclusive and ethnically diverse. The adequacy of our four-part training and support package (including trainer-led education, observations of practice, self-directed learning and clinical supervision with peer mentoring opportunities) is evident from the successful delivery of 464 health checks to working-age adults, that included brief tailored health advice and signposting based on Making Every Contact Count (MECC). 

### 4.1. IPL Outcomes in Workplace Health Promotion Practice

One hundred percent of our healthcare trainees attained the pre-determined competencies aligned with “Skills for Health” and the UK NHS Health Check Competency Framework. This study demonstrated that engaging teams of trainee healthcare professionals in workplace health promotion activities results in significant improvement in *all* areas of IPL as measured by the IRV-IPL instrument: collaboration, coordination, cooperation, communication, and commendation.

The linear increase in IPL competencies (before, after, what if) indicates not only an immediate attainment of IPL competencies, but demonstrates the increased confidence of IPL volunteers to apply these competencies to other contexts and settings. The uniqueness of this IPL activity is that it provided IPL opportunities that involved multiple healthcare disciplines (cross-disciplinary learning), as well as opportunities for learning between IPL volunteers who were registered healthcare professionals with prior experience of health promotion practice (acting as peer mentors), working alongside novices engaging in health promotion practice for the first time. Competency attainment (across before, after, what if) was evident in all of the IPL volunteers, irrespective of prior qualifications, training or experience. This demonstrates that the context (e.g., team delivery of health checks) and the setting (e.g., industry workplaces of any size) were appropriate and valuable IPL opportunities at any stage of healthcare training.

### 4.2. Theoretical Context of IPL in “Real-World” Workplace Health Promotion

Drawing on theory is important to more fully understand the nature of interprofessional education [49,50]. Socio-cultural learning theory [51] could be used as a lens to explore the team collaboration required to implement these workplace health checks. Here, the learning was situated in a “real-world” health promotion intervention in a workplace setting, with guided participation built on training and support between the project team and WHIRL team of IPL volunteers offering different levels of expertise and experience. This created a community of practice (CoP) which is the basic unit of analysis within a social learning system, defined as a “group of people who share a concern or a passion for something they do and learn how to do it better as they interact regularly” [50,51,52]. In this context, the process of participating in a community of practice with situated learning resulted in “legitimate peripheral participation”, which is a contextual social phenomenon whereby newcomers become experienced members and eventually old-timers of a community of practice or collaborative project [53]. In this context, the community of practitioners was the WHIRL health promotion team. The mastery of knowledge and skill (for pre-service healthcare students) or the application of knowledge and skill in this new non-clinical setting (for healthcare students who were already registered healthcare professionals) allowed each newcomer to the WHIRL IPL team to move from intention to learn, towards full participation in the sociocultural practice of the health check delivery. Supervisors and experienced IPL volunteers then scaffolded student “ownership” of learning through the collaborative allocation of specific areas of health check delivery, providing support and knowing how and when to intervene. 

Another relevant theoretical lens through which these findings could be interpreted is the concept of “professional identity”. One of the goals of education and training in healthcare is to facilitate and support the development of professional identity [54,55]. The delivery of the health checks involved liaison with multiple stakeholders including employers, health service providers, a research team, clinical supervisor and the IPL team of volunteers. Coupled with close teamwork within the IPL team required to deliver the health checks, these encounters helped to develop healthcare students’ sense of preparedness for practice through a community of practice, which is strongly linked to professional identity formation [56]. The sample of IPL volunteers may appear small compared to IPL studies conducted within the context of healthcare education, e.g., those IPL investigations undertaken with entire cohorts of students on their scheduled placements, or IPL views gathered from large-scale educational events. Yet, despite our IRV-IPL data being limited to 20 completions, this group of IPL volunteers was appropriate in scope, and indeed, in the context in which it was conducted a larger group was neither required nor viable. Our student team was associated with a single research study delivered across multiple industries (rather than healthcare) workplace settings, with rotating team-based health check activities undertaken outside of scheduled clinical placements, or scheduled lesson time, with multiple levels of embedded support and relationship-building through clinical supervision and peer mentoring. The diversity in this IPL sample should also be noted since it included trainees from four disciplines, different levels of study and variations in prior experience.

### 4.3. Usability, Reliability and Validity of the IRV-IPL

As a tool to assess IPL, the IRV-IPL was able to successfully measure interprofessional competencies in this workplace intervention at baseline, as an outcome of a health promotion activity (intervention outcome) and as a predictor of future competence (comparator for an alternative situation). It was practical to administer and was considered low-burden by the IPL volunteers. This tool demonstrated excellent psychometric properties. It exhibited content validity and was deemed to be an appropriate length, with postdictive and predictive validity. The tool had excellent internal consistency and reliability.

## 5. Conclusions

WHIRL IPL effectively engaged teams of healthcare trainees, peer mentors and healthcare professionals in “real-world” multi-professional workplace health promotion within the UK construction industry. This public health-focused IPL programme, delivered in a non-clinical workplace setting, demonstrably improved healthcare trainees’ interprofessional skills in all five areas of collaboration, coordination, cooperation, communication, and commendation. Research programmes are an under-utilised but excellent platform for interprofessional education resulting in competency attainment, and collaborative, practice-ready trainees for the future public health workforce. We strongly recommend the alignment of research, education and practice to maximise learning opportunities in public health. The IRV-IPL tool is a valid and reliable measure of interprofessional competencies across three scenarios; before and after health promotion activities, and as a predictor of future health promotion competence.

## Figures and Tables

**Table 1:** Competencies for WHIRL (Workplace Health InteRprofessional Learning) interprofessional learning (IPL) Volunteers.

Competencies	Description
Program Knowledge	Knowledge of the purpose, scope and aims of the Test@Work study and WHIRL IPL programme, as well as the processes and guidelines for carrying out a health check. Volunteers working in line with their own professional code of conduct or the “Code of Conduct for Healthcare Support Workers and Adult Social Care Workers”: https://www.skillsforhealth.org.uk/standards/item/217-code-of-conduct
Information governance	Three main data flows: Identifying, approaching, inviting eligible population (by Test@Work team). Recording and transferring health check data from providers to health check recipients, and evaluation forms from recipients back to the project team (by WHIRL IPL team). Data extraction from service providers for project monitoring, evaluation and quality assurance (by Test@Work Team). Volunteers demonstrate their understanding of the eligibility process. The legal requirement for those working with patient identifiable data and personal confidential data to work within the Data Protection Act (2018), the General Data Protection Regulation (GDPR, 2018) and Information Governance principles.
Employee risk assessments	Perform first line calibration on clinical equipment ready for use. Carry out the health check assessment. Undertake routine clinical measurements as allocated within the team: weight; height; body mass index (BMI), waist and waist-to-hip ratio measurements, blood pressure, screening test for mental wellbeing. Note: Volunteers facilitate transfer to service providers in the HIV point of care testing and consultation room.
Interpreting results	Agree actions to address health and wellbeing from clinical measurements. Involves the use of a risk engine together with own judgement, observations and discussion, to assess health risks. Taking into account client’s current health condition. Thereafter, understanding the results that must be communicated with them. Concerns to be raised with experienced clinical staff. Note: The HIV testing, consultation and interpretation is undertaken by independent service providers.
Brief intervention/signposting/referral	Communicate with client about health and wellbeing. This could include advice on lifestyle, alcohol, smoking, physical activity, diet, stress and mental health. Use of behaviour change methods such as motivational interviewing techniques to engaged clients in person-centred conversations about reasons for change. Risks and advice communicated in jargon-free language, tailored to client’s values and beliefs, considering wider determinants of health. Support clients to access information on services and facilities.
Communication with team	Reflection, feedback and communication of arising issues and challenges with WHIRL IPL team, Test@Work team and/or clinical supervisor.

**Table 2:** IPL constructs and application within WHIRL.

IPL Constructs		
IPL Construct	Meaning	Application within WHIRL
Collaboration	Purposeful creation of a certain outcome, and working relationships with others	Agreement of common goal, successful completion of Test@Work health check event by the IPL volunteer and project team.
Coordination	Seeks to inform other units in ensuring harmony leading towards a single direction. This explicitly emphasises awareness of the action, but not so much on the results.	Working with other team members to achieve delivery, coordinating with regards timings, rotating activities where required, and liaising with relevant project team members and external stakeholders.
Cooperation	Making contributions in a team, sharing thoughts and working together, fosters divergent thinking.	Individual contributions to team-delivered employee health checks, suggestion of ideas and improvements.
Communication	Respectfully expresses information with others for understanding. May include verbal and non-verbal strategies, as well as transmission and acquisition activities	Communicating with others delivering health checks; IPL volunteers, project team and external stakeholders as well as clients. Sharing information and teamwork.
Commendation	The appreciation of others’ competencies, accomplishments, performances, professions, roles, and identities.	Recognition of team member’s prior level of knowledge and expertise, appreciating learning and development.

**Table 3:** Inventory of Reflective Vignette (IRV)-IPL test responses (*n* = 20).

Inter-Professionalism in Learning	Before		After		What If	
	Mean	SD	Mean	SD	Mean	SD
Collaboration						
Work well with the team members	4.10	1.41	4.95	0.82	5.35	0.58
Seek others to work together	4.35	1.26	4.65	0.98	5.10	0.71
Include other team members	3.89	1.41	4.53	1.17	5.21	0.71
Use a team approach	4.05	1.14	4.65	1.08	5.10	0.78
Explain the roles/tasks	4.05	1.31	4.47	0.96	5.26	0.65
Coordination						
Negotiate tasks/responsibilities with others	4.05	1.09	4.74	0.73	5.35	0.48
Inform other participants for any changes	3.55	1.27	5.05	0.68	5.35	0.58
Work well with other groups	4.35	1.26	4.75	0.85	5.25	0.85
Discuss with others	4.25	1.20	4.80	0.89	5.20	0.69
Know the work of others	4.32	1.05	4.74	0.93	5.37	0.76
Cooperation						
Share my abilities with others	4.40	1.04	4.80	0.83	5.40	0.59
Be responsible to the team	4.45	1.05	4.85	0.93	5.45	0.68
Show my support/concern	4.42	0.90	4.50	0.92	5.26	0.56
Offer useful information	4.20	0.89	4.70	0.80	5.40	0.50
Help other participants	4.25	1.09	4.65	1.04	5.45	0.51
Communication						
Listen to others	4.55	0.99	4.74	0.93	5.21	0.71
Express my concerns	4.55	1.05	4.80	1.00	5.25	0.71
Encourage others to ask	3.80	1.36	4.47	1.02	5.20	0.76
Share my thoughts	4.45	0.94	4.80	0.89	5.35	0.58
Manage conflict	4.00	1.23	4.44	0.78	5.11	0.73
Commendation						
Give constructive feedbacks to others	3.89	0.93	4.37	0.89	5.05	0.62
Show trust in others while learning/working	4.25	1.20	4.75	0.91	5.25	0.71
Recognize the performance of others	4.10	1.02	4.65	0.74	5.25	0.63
Appreciate the contributions of others	4.35	1.13	4.60	0.99	5.40	0.59
Consider the inputs/ideas of others	4.35	1.18	4.70	1.03	5.45	0.60

Note: 1.0–1.49 Emerging; 1.5–2.49 Developing; 2.5–3.49 Minimal; 3.5–4.49 Adavanced; 4.5–5.49 Proficient; 5.5–6 Exellent.

**Table 4:** The Constructs’ Mean Comparisons (Before, After, and What If) (*n* = 20).

Constructs (*n* = 5 Items)	Before (Mean)	Before (SD)	After (Mean)	After (SD)	What If (Mean)	What If (SD)	*p* Value (Before-After/After-What If)
Collaboration	20.05	5.87	22.80	4.51	25.50	3.39	0.001/0.001
Coordination	20.30	4.52	23.60	3.11	26.25	2.86	<0.001/<0.001
Cooperation	21.70	4.23	23.05	3.90	26.70	2.43	0.04/0.001
Communication	20.95	4.19	22.35	3.68	25.60	2.98	0.03/0.001
Commendation	20.75	4.88	22.85	4.09	26.15	2.68	0.001/0.001

Note: Before, After, and What If comparisons all signficant at either the 0.05, 0.01, 0.001 alpha level.

**Table 5:** IRL-IPL reliability (*n* = 20).

Constructs (*n* = 5 Items)	Before	After	What If	Overall
Collaboration	0.92	0.92	0.86	0.94
Coordination	0.80	0.81	0.74	0.87
Cooperation	0.91	0.93	0.89	0.94
Communication	0.90	0.92	0.89	0.92
Commendation	0.95	0.96	0.90	0.96
Overall	0.96	0.97	0.96	0.97

Note: Overall refers to total scores with all related items.

**Table 6:** Relation matrix of IPL constructs to segments.

Constructs	Segments (r)		
	Before	After	What If
Collaboration			
Before	-	-	-
After	0.73	-	-
What If	0.25	0.35	-
Coordination			
Before	-	-	-
After	0.71	-	-
What If	0.43	0.52 *	-
Cooperation			
Before	-	-	-
After	0.71	-	-
What If	0.30	0.38	-
Communication			
Before	-	-	-
After	0.51 *	-	-
What If	0.25	0.37	-
Commendation			
Before	-	-	-
After	0.78	-	-
What If	0.43	0.55 *	-

Note: * Significant at 0.05 alpha level.

**Table 7:** Item-total validity (*n* = 20).

IPL Constructs and Items	Segments (r)		
	Before	After	What If
Collaboration			
Work well with the team members	0.67	0.71	0.63
Seek others to work together	0.83	0.69	0.75
Include other team members	0.78	0.71	0.65
Use a team approach	0.82	0.73	0.66
Explain the roles/tasks	0.80	0.78	0.64
Coordination			
Negotiate tasks/responsibilities with others	0.84	0.76	0.68
Inform other participants for any changes	0.04	−0.13	−0.17
Work well with other groups	0.86	0.79	0.78
Discuss with others	0.84	0.71	0.73
Know the work of others	0.81	0.89	0.70
Cooperation			
Share my abilities with others *	0.69	0.74	0.54 *
Be responsible to the team	0.83	0.84	0.62
Show my support/concern	0.90	0.83	0.84
Offer useful information *	0.75	0.81	0.55 *
Help other participants	0.82	0.83	0.69
Communication			
Listen to others	0.83	0.89	0.89
Express my concerns	0.78	0.81	0.77
Encourage others to ask	0.30	0.66	0.65
Share my thoughts	0.80	0.86	0.65
Manage conflict	0.62	0.88	0.76
Commendation			
Give constructive feedbacks to others	0.70	0.76	0.66
Show trust in others while learning/working	0.82	0.88	0.83
Recognize the performance of others	0.85	0.86	0.74
Appreciate the contributions of others	0.84	0.92	0.76
Consider the inputs/ideas of others	0.73	0.89	0.61

Note: * Significant at 0.05 alpha level.

## Supplementary Materials

The following are available online at https://www.mdpi.com/1660-4601/17/18/6815/s1, Table S1: Adapted NHS Health Check StARS framework, Table S2: Inventory of Reflective Vignette—InterProfessional Learning (IRV-IPL).

## References

[1] World Health Organization (WHO) (2010). Framework for Action on Interprofessional Education & Collaborative Practice.

[2] Bridges D., Davidson R.A., Odegard P.S., Maki I.V., Tomkowiak J. (2011). Interprofessional collaboration: Three best practice models of interprofessional education. Med. Educ. Online.

[3] Bedwell W.L., Wildman J.L., DiazGranados D., Salazar M., Kramer W.S., Salas E. (2012). Collaboration at work: An integrative multilevel conceptualization. Hum. Resour. Manag. Rev..

[4] Murray-Davis B., Marshall M., Gordon F. (2014). Becoming an interprofessional practitioner: Factors promoting the application of pre-qualification learning to professional practice in maternity care. J. Interprof. Care.

[5] Murdoch N.L., Epp S., Vinek J. (2017). Teaching and learning activities to educate nursing students for interprofessional collaboration: A scoping review. J. Interprof. Care.

[6] Dacey M., Murphy J.I., Anderson D.C., McCloskey W.W. (2010). An interprofessional service-learning course: Uniting students across educational levels and promoting patient-centered care. J. Nurs. Educ..

[7] Hanley K., Bereket S., Tuchman E., More F.G., Naegle M.A., Kalet A., Goldfeld K., Gourevitch M.N. (2018). Evaluation of the Substance Abuse Research and Education Training (SARET) program: Stimulating health professional students to pursue careers in substance use research. Subst. Abus..

[8] Chouvarda I., Mountford N., Trajkovik V., Loncar-Turukalo T., Cusack T. (2019). Leveraging interdisciplinary education toward securing the future of connected health research in europe: Qualitative study. J. Med. Internet Res..

[9] Chua A.Z., Lo D.Y., Ho W.H., Koh Y.Q., Lim D.S., Tam J.K., Liaw S.Y., Koh G.C. (2015). The effectiveness of a shared conference experience in improving undergraduate medical and nursing students’ attitudes towards inter-professional education in an Asian country: A before and after study. BMC Med. Educ..

[10] Mette M., Baur C., Hinrichs J., Oestreicher-Krebs E., Narciß E. (2019). Implementing MIA—Mannheim’s interprofessional training ward: First evaluation results. GMS J. Med. Educ..

[11] Saunders R., Dugmore H., Seaman K., Singer R., Lake F. (2019). Interprofessional learning in ambulatory care. Clin. Teach..

[12] Sharma M., Chandra P., Chaturvedi S. (2015). Team OSCE: A teaching modality for promotion of multidisciplinary work in mental health settings. Indian J. Psychol. Med..

[13] Gilfoyle E., Koot D.A., Annear J.C., Bhanji F., Cheng A., Duff J.P., Grant V.J., St George-Hyslop C.E., Delaloye N.J., Kotsakis A. (2017). Improved clinical performance and teamwork of pediatric interprofessional resuscitation teams with a simulation-based educational intervention. Pediatr. Crit. Care Med..

[14] Granheim B.M., Shaw J.M., Mansah M. (2018). The use of interprofessional learning and simulation in undergraduate nursing programs to address interprofessional communication and collaboration: An integrative review of the literature. Nurse Educ. Today.

[15] O’Shea M.-C., Reeves N.E., Bialocerkowski A., Cardell E. (2019). Using simulation-based learning to provide interprofessional education in diabetes to nutrition and dietetics and exercise physiology students through telehealth. Adv. Simul..

[16] Fitzgerald S.N., Leslie K.F., Simpson R., Jones V.F., Barnes E.T. (2018). Culturally effective care for refugee populations: Interprofessional, interactive case studies. MedEdPORTAL.

[17] Haley J., McCall R.C., Zomorodi M., De Zerdan L.S., Moreton B., Richardson L. (2019). Interprofessional collaboration between health sciences librarians and health professions faculty to implement a book club discussion for incoming students. JMLA.

[18] van Lierop M., van Dongen J., Janssen M., Smeets H., van Bokhoven L., Moser A. (2019). Jointly discussing care plans for real-life patients: The potential of a student-led interprofessional team meeting in undergraduate health professions education. Perspect. Med. Educ..

[19] Morphet J., Cant R., Hood K., Baulch J., Gilbee A., Sandry K. (2014). Teaching teamwork: An evaluation of an interprofessional training ward placement for health care students. AMEP.

[20] Vanderzalm J., Hall M.D., McFarlane L.-A., Rutherford L., Patterson S.K. (2013). Fostering Interprofessional Learning in a Rehabilitation Setting: Development of an Interprofessional Clinical Learning Unit. Rehabil. Nurs..

[21] Kent F., Keating J.L. (2015). Interprofessional education in primary health care for entry level students—A systematic literature review. Nurse Educ. Today.

[22] Christensen M., Burau V., Ledderer L. (2018). How intersectoral health promotion changes professional practices: A case study from Denmark. Health Promot. Pract..

[23] Grace S., Coutts R. (2017). An interprofessional health assessment program in rural amateur sport. J. Interprof. Care.

[24] Diwan S., Perdue M., Lee S.E., Grossman B.R. (2016). Health promotion practice and interprofessional education in aging: Senior wellness fairs. Gerontol. Geriatr. Educ..

[25] Lee S., Blake H., Lloyd S. (2010). The price is right: Making workplace wellness financially sustainable. Int. J. Workplace Health Manag..

[26] Blake H., Zhou D., Batt M.E. (2013). Five-year workplace wellness intervention in the NHS. Perspect. Public Health.

[27] Malik S.H., Blake H., Suggs L.S. (2014). A systematic review of workplace health promotion interventions for increasing physical activity. Br. J. Health Psychol..

[28] Poscia A., Moscato U., La Milia D.I., Milovanovic S., Stojanovic J., Borghini A., Collamati A., Ricciardi W., Magnavita N. (2016). Workplace health promotion for older workers: A systematic literature review. BMC Health Serv. Res..

[29] White M., Dionne C., Wärje O., Koehoorn M., Wagner S., Schultz I., Koehn C., Williams-Whitt K., Harder H., Pasca R. (2016). Physical activity and exercise interventions in the workplace impacting work outcomes: A stakeholder-centered best evidence synthesis of systematic reviews. Int. J. Occup. Environ. Med..

[30] Brand S.L., Coon J.T., Fleming L.E., Carroll L., Bethel A., Wyatt K. (2017). Whole-system approaches to improving the health and wellbeing of healthcare workers: A systematic review. PLoS ONE.

[31] Jirathananuwat A., Pongpirul K. (2017). Promoting physical activity in the workplace: A systematic meta-review. J. Occup. Health.

[32] Skamagki G., King A., Duncan M., Wåhlin C. (2018). A systematic review on workplace interventions to manage chronic musculoskeletal conditions. Physiother. Res. Int..

[33] Tarro L., Llauradó E., Ulldemolins G., Hermoso P., Solà R. (2020). Effectiveness of workplace interventions for improving absenteeism, productivity, and work ability of employees: A systematic review and meta-analysis of randomized controlled trials. Int. J. Environ. Res. Public Health.

[34] Blake H. How to Manage Employee Wellbeing in a Small Business. https://www.financedigest.com/how-to-manage-employee-wellbeing-in-a-small-business.html.

[35] Blake H., Hussain B., Hand J., Rowlands D., Juma A., Evans C. (2018). Employee perceptions of a workplace HIV testing intervention. Int. J. Workplace Health Manag..

[36] Blake H., Hussain B., Hand J., Juma A., Evans C. (2019). Employers’ views of the “Healthy Hub Roadshow”: A workplace HIV testing intervention in England. Aids Care.

[37] Blake H., Banerjee A., Evans C. (2018). Employer attitudes towards general health checks and HIV testing in the workplace. Public Health.

[38] Green B.N., Johnson C.D. (2015). Interprofessional collaboration in research, education, and clinical practice: Working together for a better future. J. Chiropr. Educ..

[39] Ong I.L., Diño M.J.S., Calimag M.M.P., Hidalgo F.A. (2019). Development and validation of interprofessional learning assessment tool for health professionals in continuing professional development (CPD). PLoS ONE.

[40] MacDonald C.J., Archibald D., Trumpower D., Casimiro L., Cragg B., Jelley W. (2010). Designing and Operationalizing a Toolkit of Bilingual Interprofessional Education Assessment Instruments. JRIPE.

[41] Bottenberg M.M., DeWitt J.E., Wall G.C., Fornoff A., Stelter N., Soltis D., Eastman D.K. (2013). Assessment of interprofessional perceptions and attitudes of health professional students in a simulation laboratory setting. Curr. Pharm. Teach. Learn..

[42] Public Health England (2020). NHS Health Check StARS Framework: A Systematic Approach to Raising Standards. https://www.healthcheck.nhs.uk/commissioners-and-providers/delivery/nhs-health-check-stars-framework/.

[43] Kirkpatrick D.L., Kirkpatrick J.D. (2006). Evaluating Training Programs: The Four Levels.

[44] Ruzafa-Martinez M., Lopez-Iborra L., Moreno-Casbas T., Madrigal-Torres M. (2013). Development and validation of the competence in evidence based practice questionnaire (EBP-COQ) among nursing students. BMC Med. Educ..

[45] Brown R., Hewstone M. (2005). An integrative theory of intergroup contact. Advances in Experimental Social Psychology.

[46] Roets A., Kruglanski A.W., Kossowska M., Pierro A., Hong Y. (2015). The motivated gatekeeper of our minds. Advances in Experimental Social Psychology.

[47] Allport G.W., Clarke K., Pettigrew T. (1954). The Nature of Prejudice, Unabridged, 25th Anniversary Ed..

[48] Blake H., Somerset S., Evans C. (2020). Development and fidelity testing of the test@ work digital toolkit for employers on workplace health checks and Opt-In HIV testing. Int. J. Environ. Res. Public Health.

[49] Reeves S., Hean S. (2013). Why we need theory to help us better understand the nature of interprofessional education, practice and care. J. Interprof. Care.

[50] Roberts C., Kumar K. (2015). Student learning in interprofessional practice-based environments: What does theory say?. BMC Med. Educ..

[51] McInerney D.M., Walker R.A., Liem G.A.D. (2011). Research on Sociocultural Influences on Motivation and Learning.

[52] Wenger E. (2000). Communities of practice and social learning systems. Organization.

[53] Lave J., Wenger E. (1991). Situated Learning: Legitimate Peripheral Participation.

[54] Cruess R.L., Cruess S.R., Boudreau J.D., Snell L., Steinert Y. (2014). Reframing medical education to support professional identity formation. Acad. Med..

[55] Wald H.S. (2015). Professional identity (Trans)formation in medical education: Reflection, relationship, Resilience. Acad. Med..

[56] Cruess S.R., Cruess R.L., Steinert Y. (2019). Supporting the development of a professional identity: General principles. Med. Teach..

