# Supplementary material for: WHIRL Study: Workplace Health Interprofessional Learning in the Construction Industry
Source: Int J Environ Res Public Health. 2020 Sep 18;17(18):6815. doi: 10.3390/ijerph17186815 (PMC7558403; doi:10.3390/ijerph17186815)
Supplement: Supplementary file 1 [file ijerph-17-06815-s001.pdf]

Table S1. Adapted NHS Health Check StARS framework.

Section 1: Vision and Leadership		Source of Evidence
1.a	Does the funder receive reports or dashboard indicators on the Health Check programme?	Quarterly funder report.<br>Final report to institutional IPL team.
1.b	Does the project lead assert a clear ambition for the Health Check programme?	Ambition stated in study protocol: https://clinicaltrials.gov/ct2/show/NCT04292002?term=holly+blake&draw=2&rank=5<br>ClinicalTrials.gov Identifier: NCT04292002
1.c	Do you have a vision for delivering the Health Check programme that is widely understood and shared by others across the organization(s)?	Commitment to support the implementation is set out in the delivery or business plans of service providers and participating organisations. Additional IPL plans outlined in news bulletin and email.
1.d	Have members recently conducted a local scrutiny enquiry that includes the Health Check?	Quarterly project monitoring.<br>Health check quality overseen by clinical collaborators (nurses/paramedic).
1.e	Are there clinical leadership champions engaged with the Health Check programme?	Test@Work Steering Group includes 4 clinical champions.
1.f	Have you provided briefing on the Health Checks in the last 6 months?	Quarterly funder report.
1.g	Has your local delivery of the programme been championed at a PHE centre level?	Letter of support for Test@Work study from PHE Public Health Consultant.<br>Poster presentation accepted at national HIV England event.
1.h	Has the project lead and team (twice a year or more) highlighted the Health Check to prospective corporate participants?	Email correspondence<br>Business Network Presentations<br>Ingenuity Network events<br>Trade publications (e.g. Employee Benefits; The Construction Index, UK - 81,300 readers and 2.322 million website visits in 2019.
1.i	Does your team act as an exemplar in supporting staff to access a Health Check?	Health check event volunteers are provided with time to have a health check.
1.j	Are your offers on track to achieve 20% this year?	Project uptake of HIV test (focus of Test@Work) as a % of health check participants always exceeds this.

1.k	Have you achieved year-on-year improvement in uptake? (aspiring to 75%)?	Increase in uptake from project year 1-2.<br>Exceeds aspiration of 75%.

Section 2: Planning and commissioning		Source of evidence
2.a	i) Do you have a current Health Check delivery plan?	Programme plan is in the study protocol: https://clinicaltrials.gov/ct2/show/NCT04292002?term=holly+blake&draw=2&rank=5 ClinicalTrials.gov Identifier: NCT04292002 Delivery and Action plans with materials are provided in IPL training sessions and by email.
	ii) If so, does your plan include Specific, Measurable, Achievable, Realistic, Timebound objectives which are monitored regularly?	Yes. Receipt of funds is dependent on quarterly achievement of project milestones, monitored by project lead and funder.
	iii) Does your project lead sign the plan off?	Yes. Delivery plan was developed by project lead in collaboration with clinical team.
	iv) Does the funder sign off the plan?	Yes. Content and delivery plans were outlined and reviewed/approved at the funding application stage.
	v) Can you demonstrate improvement against the objectives in your own plan?	Yes. Quarterly project monitoring demonstrated targets are met. Process evaluation is conducted to evaluate health check delivery. Embedded WHIRL study demonstrates attainment of competencies for health check volunteers.
	vi) Is there a clear connection between the Health Check plan, and health and wellbeing strategy?	Systems approach taken to ensure standards are met (mapping to NHS Health Check StARS Framework) Aligns with NICE Pathway for Workplace Health (2019) Local alignment with corporate health and wellbeing or corporate social responsibility programmes.
2.b	Do you have dedicated resources to deliver the programme as planned?	Test@Work study is funded by Gilead Sciences, Inc. (Grant Reference Number - H. Blake - INUK276 5347HIVDVE). Budget allows delivery of the programme as planned. Embedded WHIRL study is supported by SBEA Award to project lead.
2.c	How do you monitor expenditure on the Health Check programme?	Expenditure on the programme is reported as part of the requirement for the Gilead Sciences, Inc. and SBEA grants. Budget is monitored by project lead.

2.d	Do you have a service specification for commissioned providers?	Service specification agreed with commissioned HIV testing providers.
2.e	To what extent do your provider contracts include output, quality and outcome standards?	Service level agreement with commissioned HIV testing providers.
2.f	Has there been an internal audit or monitoring of the programme?	Programme is monitored weekly by project lead, with bi-monthly team meetings. Quarterly reporting to funder. Embedded WHIRL study assesses attainment of competencies in health checks team.<br>Final report will include recommendations.

Section 3: Partnerships		Source of evidence
3.a	Do you have active partnerships which contribute to local plans for the delivery of the Health Check programme?	Collaboration with corporate partners to determine site delivery plans. Health checks delivered as per protocol. Local delivery issues addressed within team or referred to steering group.
3.b	i) Do you have a Health Check steering group?	Embedded WHIRL project team are the Test@Work health check steering group.
	ii) How does the steering group engage a range of partners i.e. local elected members, local authority officers, representatives from the CCG, key groups in the local voluntary sector, PHE centres and local communities?	As in 3a. Direct email/phone contact with corporate partners, delivery partners (service providers). Stakeholder consultation and stakeholder dissemination includes key groups (clinical, occupational health, trade union, human resources, service users).
	iii) Does the work of the group have clear links to a health and wellbeing board?	Project lead sits on strategic workforce health and wellbeing boards. Final report will be sent to PHE Public Health Consultant.
3.c	Do you have regular links and communications with CCGs and GPs, outside of the context of a steering group?	As in 3.b ii). CCGs/GPs/PHE Public Health Consultants invited to stakeholder dissemination at project end, project summary will be provided for newsletters and dissemination.
3.d	Can you evidence that partners have worked together in securing local achievements?	SBEA – business engagement award to project lead.<br>Case for partnership working with service providers<br>Email correspondence with partners<br>Wellbeing awards to corporate partners
3.e	Would you agree that the Health Check is visible in other plans and objectives, policy and procedures across internal and external partners?	Commitment to support the implementation is set out in the delivery or business plans of service providers, and participating organisations in the private sector.
3.f	Do you contribute to and benefit from relevant existing sub-national (PHE Region) networks?	Test@Work project summary will be provided to PHE networks.

3.g	Can you show instances where you have collaborated with people outside of the local authority to improve your service (e.g. through cross-boundary working arrangements with neighbours)?	Project involves cross-boundary working with service providers from multiple geographical regions<br>Agreement with corporate partners to offer Health Checks to non-employees working on study sites (e.g. self-employed contractors, agency workers)

Section 4: Service delivery<br>4.1 Identification and invitation		Source of evidence
4.1.a	How do you identify the eligible organisations?	Organisation eligibility determined by geographical location: regions with agreed PHE commissioner governance
4.1.b	Is a systematic strategy used to identify the eligible local population that will be invited for a Health Check?	Worker eligibility as per study protocol<br>Agreement with host organisations regarding access for non-employees working on study sites (e.g. self-employed contractors, agency workers).<br>Communicated via direct emails/phone and provider process training.
4.1.c	Where individuals are sent an invitation letter, does this contain information on the contact details of the organisation providing the Health Check, set out how their data will be handled, and provide information on the harms and benefits of having an NHS Health Check?	Host organisations invited by project team.<br>Worker invitation circulated by participating organisation.<br>Participant information sheets provided with contact details.<br>Verbal explanations from service providers.<br>Provider process training.
4.1.d	Is there an agreed protocol that requires at least one follow-up with non-responders or people that do not attend a Health Check?	Organisations agreeing to host events followed up minimum of 2 times.<br>Workers that do not attend booked health check appointment can re-book or attend drop-in without booking.
4.1.e	What systems are in place to provide Health Checks to people who request them and to people who are not registered with a GP?	Test@Work health checks open to all workers accessing the sites on request or drop-in, including those not registered with a GP.<br>Signposting to further health services is provided, includes third sector/charities. Provider process and MECC training.

4.2 Risk assessment		Source of evidence
4.2.a	How do you ensure that a complete Health Check, for those accepting the offer, is undertaken and recorded in line with quality standards?	Standardised record forms (Ethics Ref: LT12042016). Provider process training. Project governance and data monitoring. Provider governance processes for HIV tests and consultation (e.g. local CCGs).
4.2.b	Do you use a cardiovascular risk calculator in line with NICE guidance?	Included in worker health check pack and signposting.
4.2.c	How do you ensure that individual risk factor measures are communicated effectively and recorded in line with quality standards?	Tailored advice and signposting is provided at all checks in line with best practice guidance. Provider process and MECC training, provider support materials.
4.2.d	How do you ensure that equipment calibration and incident reporting is undertaken as set out in quality standards?	Regular calibration of equipment by health promotion coordinator.
4.2.e	Are you implementing risk assessment and quality control practices?	Lone worker risk assessments for health promotion team. 100% data checking for record quality control. Risk assessment and quality control for HIV point of care testing responsibility of service provider. Associated clinical governance in place for providers.
4.2.f	Have quality assurance visits been undertaken with providers in the last 12 months to ensure that checks are being delivered in line with best practice guidance?	Quality assurance is undertaken within the Test@Work management team. 100% of health check record sheets are checked by project coordinator. Management team member attends >90% of events. Content and delivery of checks is overseen by clinical steering group members in WHIRL team.

4.3 Risk management and follow-up		Source of evidence
4.3.1.a	How do you ensure that individuals with abnormal risk profiles receive appropriate follow-up in line with the best practice guidance?	Tailored advice and signposting is provided at all checks in line with best practice guidance. Provider MECC training and support materials. Worker health check pack provided. HIV test follow-up referrals are made by service providers as required. Evaluation of user experience and understanding.
4.3.1.b	How do you ensure that individuals with abnormal risk profiles receive appropriate clinical management in line with the best practice guidance?	As in 4.3.1.a
4.3.1.c	Where a statin, antihypertensive or other clinical treatment is offered how do you ensure that this is recorded along with whether or not it is accepted?	Research protocol does not include treatment. As in 4.3.1.a
4.3.1.d	Are individual's at high risk of CVD managed in accordance with NICE guidelines?	Recommendation to visit GP. Tailored advice and worker health check pack and signposting. Provider MECC training and support materials.
4.3.2.a	Do you have evidence that where clinically appropriate, all individuals who have a check are given lifestyle advice, regardless of their CVD risk score, to help them manage and reduce their CVD risk?	Tailored lifestyle advice and signposting (where appropriate) is verbally provided for all health check participants. Worker health check pack provided. Provider MECC training and support materials.
4.3.2.b	Can providers refer into a range of lifestyle programmes that address the individual's modifiable risk factors?	Signposting to online support and health services. Provider MECC training and support materials.
4.3.2.c	Are these lifestyle services based on the latest NICE guidelines?	Yes. As in 4.3.2b.
**Section 5: Competence, training and development**		**Source of evidence**

5.a	How do you ensure that providers meet the core competences? (source: NHS Health Check competence framework; standard 6 QS framework pg21)	Screening to join Test@Work delivery team. Provider MECC training, observations and on-the-job training with clinical team member. Embedded WHIRL study delivery volunteers complete IPL competency and reflective exercise. Evidenced competency progression. Participating organisations are provided with line manager toolkit (about health checks and HIV testing).
5.b	Have delivery staff recently been offered brief intervention training?	Yes. Health check delivery team have attended MECC brief intervention training and process training. Additional on-the-job training with clinical team member.
5.c	Are Health Check training and education materials available for health professionals?	Health check delivery team provided with resource pack with guidance and signposting materials.
5.d	How are provider's experiences used to inform future training requirements?	Every provider and WHIRL volunteer completes a post-event evaluation form. Interviews conducted with host organisations.
5.e	How are patient's experiences used to inform future training requirements	Every health check recipient completes a post-event evaluation form. Interviews conducted with workers.

Section 6: Information governance and data<br>6.1 Data recording		Source of evidence
6.1.a	How do you ensure a consistent approach to recording information on non-responders, people that opt-out and DNA, is adopted in line with quality standard 2? (source: standard 2 QS framework pg15)	Standard data collection form. Provider training. Record non-attendance and opt-out (with reasons for decline). Test results not stored at individual level by project team; no DNA stored by the project team. Service level agreement with providers.
6.1.b	How do you ensure that all providers record data appropriately?	Standard data collection form. Provider training. Research team completes Research Integrity and Good Clinical Practice training.<br>Adhere to GDPR.
6.1.c	How do you ensure that the completion and outcome of a Health Check e.g. signposting/referral to local lifestyle services is recorded?	Primary aim of study is to determine reach and uptake, rather than outcome. Tailored advice and signposting provided, standardised resources for providers, and standard take-away materials for workers.
6.1.d	How do you ensure that providers of lifestyle services record information on individuals' outcomes following the completion of an intervention?	Standard data collection form. Service providers store HIV test uptake and results in line with governance requirements (Service Level Agreement).
6.1.e	How do you ensure that GPs routinely upload data from a Health Check, provided by an alternative provider, onto the patient's record? (programme standards specifies within two days)	n/a - not a NHS Health Check.<br>Health check data is provided directly to recipient, is not stored by the study team and is not provided to GPs or employers.

	6.2 Information governance	Source of evidence
6.2.a	Is there evidence that data processers (GPs or organisations acting on their behalf and other providers) are compliant with the Information Commissioners level 2 toolkit?	n/a - See 6.1.e<br>Service Level Agreement with HIV testing provider
6.2.b	Where a third party data processer is identifying the eligible population and/or sending invitations on behalf of a data controller e.g. general practice, is there evidence of a current data processing contract between the data controller and the data processor?	n/a - See 6.1.e<br>Service Level Agreement with HIV testing provider
6.2.c	Where the National Health Authority Information System is used to identify the eligible population is there a data processing contract between the data controller (NHS England) and the data processor?	n/a – eligibility of health check participants is outlined in protocol and all workers at host sites are eligible.

	6.3 Data return and monitoring	Source of evidence
6.3.a	Do you have evidence that data on offered and received NHS Health Checks is reported to PHE in line with the single data list returns and on time?	n/a not NHS Health Check. Data on offered and received health checks is collated by project coordinator and reported to Chief Investigator weekly, and quarterly to funder as per project reporting requirement.
6.3.b	Do commissioners receive anonymous information from providers above and beyond offers and uptake?	Anonymous data on HIV testing uptake and outcomes (only) is provided directly to commissioners by service providers. See 6.1.e for remaining data.
6.3.c	Do you monitor the proportion of individuals recalled in five years, if they remain eligible?	n/a - one-off workplace health checks with no follow-up, as per protocol.
6.3.d	Do you monitor how your local implementation compares to other similar areas?	Few employers offer general health testing for employees, opt-in HIV testing is exceptionally rare (Blake et al., 2018). Systematic review underway will allow comparisons.
6.3.e	Do you provide quarterly internal performance reporting on the delivery of the programme?	Quarterly reporting to funder. Monthly reporting within the team. Weekly reporting to Chief Investigator.
6.3.f	Where the Health Check is not conducted by general practice is there confidential and timely transfer of patient identifiable data back to their GP?	No patient identifiable data is stored by project team.
6.3.g	Do you have a protocol in place on the management and information-sharing of any Serious Untoward Incidents (SUIs) in delivery of Health Checks?	Yes - study protocol (Ethics Ref: LT12042016).

Section 7: Communication		Source of evidence
7.a	Do you have a communication/ marketing plan and an approach for engaging with key stakeholders?	Project promotion and dissemination plan in place. Communication to workers relating to health checks is via host organisations.
7.b	Do you link with and amplify national and/or regional (PHE centre) marketing campaigns?	Link to national campaigns through tailored advice and worker health check resource pack.
7.c	How do you make use of internal communication channels in ensuring and improving quality and uptake of Health Checks?	Weekly updates to Chief Investigator. Monthly team meetings. Quarterly reporting. Emails and virtual meetings (Microsoft Teams). Debrief with delivery partners, evaluation forms and feedback channels. Formal assessment of volunteer competencies through embedded WHIRL project with reflection and feedback. Liaison (email, telephone, face-to-face) with host sites regarding venues and processes.
7.d	How do you make use of external communication channels in ensuring and improving quality and uptake of Health Checks?	Email correspondence (via host sites)<br>Business Network Presentations<br>Ingenuity Network events<br>Trade publications (e.g. Employee Benefits; The Construction Index, UK - 81,300 readers and 2.322 million website visits in 2019.<br>Participating organisations are provided with line manager toolkit (about health checks and HIV testing).
7.e	How do you engage with voluntary, community or professional bodies to raise awareness of the programme among the public?	Third sector delivery partners.<br>Embedded WHIRL project with volunteer delivery team of healthcare trainees.<br>Promotion via community groups.<br>Trade publications.
7.f	Can you show instances where you have spent money on marketing and communication?	Privacy screen with project promotion.<br>Stakeholder consultation events.<br>Dissemination costs – open access journal publishing fees.

7.g	Are tried and tested branding and marketing materials used to promote the programme?	Institutional branding.
7.h	Is there an entry for the service on the NHS Choices directory?	n/a funded project with health check intervention - not a service

Section 8: Programme development and evaluation		Source of evidence
8.a	How do you invite feedback from individuals on their experience of the Health Check e.g. on such as i.e. location, time, number of appointments, provider etc, in line with quality standards?	Post-check worker evaluation form<br>Post-check worker interview<br>Post-check service provider evaluation form<br>Host organisation - line manager interviews
8.b	Have you used data from users to influence or change the design and delivery of the service?	Health check delivery is per protocol.<br>Data collected at stakeholder consultation informed the line manager toolkit.<br>Feedback from participants informs minor adjustments to room layout, venue etc.
8.c	Have you evaluated how successful the service is at helping patients to understand their CVD risk?	Interviews with workers (health check attendees).
8.d	Have you undertaken activity to better understand local public attitudes or behaviour towards the Health Check programme?	Formal mixed-methods evaluation, including host organisations, service providers and workers.
8.e	Do you work with providers to monitor issues or challenges arising in delivery of Health Checks?	Issues and challenges raised by service providers or host organisations are managed by the project team onsite or Chief Investigator as appropriate.<br>Challenges raised by WHIRL volunteers are managed onsite by the supervising clinical team member.
8.f	Have you, or are you, collecting evaluation data on the outcomes of your programme?	As in 8.d

Section 9: Innovation		Source of evidence
9.a	Do you have systems in place for learning from local innovative delivery?	Formal evaluation to generate insights. Formal dissemination plan to include lay summary, scientific journal articles, conferences targeting academics, public health specialists, commissioner and policy makers.
9.b	Have you used technology in different ways to support delivery?	Remote health promotion for workers: opt-in SMS messaging for 10-weeks post event.<br>Digital toolkit for line managers providing guidance on health checks and HIV testing
9.c	Have you developed innovative solutions to meet local needs?	Set-up adjusted according to host site preference and facilities. Engagement of additional staff (WHIRL volunteers) to increase participant numbers at any one time, and generate interprofessional learning opportunities for healthcare trainees.

Section 10: Equity and health inequality		Source of evidence
10.a	Do you monitor your local data on equitable uptake of the Health Check, looking at uptake among the disadvantaged and where need is likely to be greatest?	Rationale for target employee population (industry with identified need). Monitoring data and examination of uptake is a primary outcome. Results will inform future provision.
10.b	Is Health Check information available in other formats (Braille, languages, easy read).	Not designed for this project due to funding limits; special requests could be proposed to study team. Resource packs in English. Language alternatives available (majority of project team multi-lingual).
10.c	Have the needs of all communities, including the disadvantaged and those sharing a protected characteristic, informed both the commissioning and delivery of the programme?	Where possible given funding limits. Checks were free to access. Line manager toolkit, resource packs and training materials reviewed by Equality, Diversity and Inclusion representative.

Table S2. INVENTORY OF REFLECTIVE VIGNETTE – INTERPROFESSIONAL LEARNING (IRV-IPL).

**IRV-IPL**	**BEFORE the Test@Work health checks I was able to:**						**AFTER the Test@Work health checks I was able to:**						**If I did health checks in the future I would be able to:**					
**Collaboration: purposeful working relationships**	**1**	**2**	**3**	**4**	**5**	**6**	**1**	**2**	**3**	**4**	**5**	**6**	**1**	**2**	**3**	**4**	**5**	**6**
Work well with the team members																		
Seek other members to accomplish the work																		
Include other team members in making plans/decisions																		
Use a team approach to achieve the goals/outcomes																		
Explain the roles/tasks of each team member																		
**Coordination: faithful mutual performances**	**1**	**2**	**3**	**4**	**5**	**6**	**1**	**2**	**3**	**4**	**5**	**6**	**1**	**2**	**3**	**4**	**5**	**6**
Negotiate tasks/responsibilities with other team																		

members																		
Inform others for any changes																		
Work well with team members doing other tasks																		
Discuss your plans/actions with other team members																		
Know the work/responsibility of other team members																		
**Cooperation: helpful personal interactions**	**1**	**2**	**3**	**4**	**5**	**6**	**1**	**2**	**3**	**4**	**5**	**6**	**1**	**2**	**3**	**4**	**5**	**6**
Share my inputs/abilities with other team members																		
Be responsible with my contributions to the team																		
Show my support/concern for other team members																		
Offer useful																		

information to other participants																		
Help other team members when necessary																		
**Communication: respectful social interactions**	**1**	**2**	**3**	**4**	**5**	**6**	**1**	**2**	**3**	**4**	**5**	**6**	**1**	**2**	**3**	**4**	**5**	**6**
Listen actively to other team members																		
Express my concerns in a professional manner																		
Encourage others to ask useful questions politely																		
Share my thoughts in a clear effective manner																		
Manage conflict in a courteous manner																		
**Commendation: thoughtful professional appreciations**	**1**	**2**	**3**	**4**	**5**	**6**	**1**	**2**	**3**	**4**	**5**	**6**	**1**	**2**	**3**	**4**	**5**	**6**
Give constructive																		

feedbacks to other team members																		
Show trust in other team members while learning/working																		
Recognize the performance of other participants																		
Appreciate the contributions of other team members																		
Consider the inputs/ideas of other participants																		

For each statement below (before, after and future) please tick the numerical answer that describes your level of performance as follows:.1 = Emerging; 2 = Developing; 3 = Minimal; 4 = Proficient; 5 = Advanced; 6 = Excellent.
